# EMID2 is a novel biotherapeutic for aggressive cancers identified by in vivo screening

**DOI:** 10.1186/s13046-023-02942-4

**Published:** 2024-01-10

**Authors:** Ambra Cappelletto, Edoardo Alfì, Nina Volf, Thi Van Anh Vu, Francesca Bortolotti, Giulio Ciucci, Simone Vodret, Marco Fantuz, Martina Perin, Andrea Colliva, Giacomo Rozzi, Matilde Rossi, Giulia Ruozi, Lorena Zentilin, Roman Vuerich, Daniele Borin, Romano Lapasin, Silvano Piazza, Mattia Chiesa, Daniela Lorizio, Luca Triboli, Sandeep Kumar, Gaia Morello, Claudio Tripodo, Maurizio Pinamonti, Giulia Maria Piperno, Federica Benvenuti, Alessandra Rustighi, Hanjoong Jo, Stefano Piccolo, Giannino Del Sal, Alessandro Carrer, Mauro Giacca, Serena Zacchigna

**Affiliations:** 1 Cardiovascular Biology, International Centre for Genetic Engineering and Biotechnology (ICGEB), Trieste, Italy; 2 Department of Life Sciences, University of Trieste, Trieste, Italy; 3 Veneto Institute of Molecular Medicine, Padova, Italy; 4 University of Padova, Padova, Italy; 5 Molecular Medicine, International Centre for Genetic Engineering and Biotechnology (ICGEB), Trieste, Italy; 6 Department of Engineering and Architecture, University of Trieste, Trieste, Italy; 7 Bioinformatics, International Centre for Genetic Engineering and Biotechnology (ICGEB), Trieste, Italy; 8 Bioinformatics Facility, Department of Cellular, Computational and Integrative Biology - CIBIO, University of Trento, Trento, Italy; 9 Centro Cardiologico Monzino, Milano, Italy; 10 Cancer Cell Signaling, International Centre for Genetic Engineering and Biotechnology (ICGEB), Trieste, Italy; 11 Wallace H. Coulter Department of Biomedical Engineering, Emory University, Georgia Institute of Technology, Atlanta, GA USA; 12 Tumor Immunology Unit, Department of Sciences for Health Promotion and Mother-Child Care “G. D’Alessandro”, University of Palermo, Palermo, Italy; 13 Histopathology Unit, Institute of Molecular Oncology Foundation (IFOM), ETS - The AIRC Institute of Molecular Oncology, Milan, Italy; 14 Pathology Department Azienda Sanitaria Universitaria Giuliano-Isontina and University of Trieste, Trieste, Italy; 15 Cellular Immunology, International Centre for Genetic Engineering and Biotechnology (ICGEB), Trieste, Italy; 16 IFOM ETS, The AIRC Institute of Molecular Oncology, Milan, Italy; 17 King’s College London, British Heart Foundation Centre of Research Excellence, London, UK; 18 Department of Medicine, Surgery and Health Sciences, University of Trieste, Trieste, Italy

**Keywords:** In vivo screening, Cancer, Cell invasiveness, AAV vectors, Gene therapy, Biotherapeutics

## Abstract

**Background:**

New drugs to tackle the next pathway or mutation fueling cancer are constantly proposed, but 97% of them are doomed to fail in clinical trials, largely because they are identified by cellular or in silico screens that cannot predict their in vivo effect.

**Methods:**

We screened an Adeno-Associated Vector secretome library (> 1000 clones) directly in vivo in a mouse model of cancer and validated the therapeutic effect of the first hit, EMID2, in both orthotopic and genetic models of lung and pancreatic cancer.

**Results:**

EMID2 overexpression inhibited both tumor growth and metastatic dissemination, consistent with prolonged survival of patients with high levels of EMID2 expression in the most aggressive human cancers. Mechanistically, EMID2 inhibited TGFβ maturation and activation of cancer-associated fibroblasts, resulting in more elastic ECM and reduced levels of YAP in the nuclei of cancer cells.

**Conclusion:**

This is the first in vivo screening, precisely designed to identify proteins able to interfere with cancer cell invasiveness. EMID2 was selected as the most potent protein, in line with the emerging relevance of the tumor extracellular matrix in controlling cancer cell invasiveness and dissemination, which kills most of cancer patients.

**Supplementary Information:**

The online version contains supplementary material available at 10.1186/s13046-023-02942-4.

## Background

Over 90% of drug development fails despite the use of state-of-the-art screening and optimization strategies [1]. This percentage is even higher for anti-cancer drugs, which fail in advancing to clinical use because of problems with efficacy or toxicity in 97% of cases [2]. Thus, more stringent and advanced screening methods are required to verify the therapeutic potential of new drugs.

Here, we exploit an innovative selection strategy, developed by our laboratory, to screen genetic libraries for molecules able to modulate cancer cell invasiveness directly in vivo (herein referred to as CanSel, for Cancer Selection). CanSel is based on the delivery of an arrayed library of cDNAs cloned in Adeno-Associated Vectors (AAV) in a relevant animal model, followed by the induction of a selective stimulus that eliminates most of the transduced cells. Should any of the transgenes exert a protective effect against the selecting stimulus, it will result enriched. This in vivo approach overcomes the major limitations of cellular screening, as shown in our previous work, where we exploited it to discover novel molecules protecting from cardiac ischemia [3, 4], and enhancing the engraftment of mesenchymal stromal cells for tissue repair [5]. Here, we adapted this strategy to screen for the most potent proteins inhibiting cancer cell invasiveness.

Traditional cancer treatments mainly inhibit the proliferation of cancer cells, using either radiotherapy or chemotherapy. Monoclonal antibodies, targeting cancer-specific receptors, and immunotherapy, including chimeric antigen receptor (CAR) T cells, have significantly prolonged the survival of patients with aggressive cancers. However, a limited number of patients respond to these treatments and most of them become resistant, often experiencing refractory disease within a year [6–8]. Therapeutic strategies aiming at interfering with the invasiveness of cancer cells, which eventually leads to their dissemination and patient death, are still missing.

A growing body of evidence indicates that both cellular and extracellular elements modify tumor development, dissemination and response to therapy [9]. Mechanical properties of the tumor extracellular matrix (ECM), such as stiffness and viscosity, also influence oncogene activation, cell morphology and migration, as well as the efficacy of existing therapies [9, 10]. However, therapeutic interventions specifically targeting cancer ECM still fall short of expectations [11].

Thus, we screened for secreted proteins able to shape the extracellular milieu to inhibit cancer cell invasiveness. Being secreted, the selected proteins could be delivered exogenously as biotherapeutics, avoiding the hurdles of gene therapy. In this way, we identified EMI domain-containing protein 2 (EMID2) as the best hit in dampening cancer cell invasiveness. We proved that EMID2 overexpression at the site of primary tumors reduces its growth and dissemination at distant sites, using mouse models of orthotopic and genetic tumors, relevant for human cancer. We also showed that high levels of EMID2 are protective in highly aggressive human cancers.

## Methods

Detailed experimental data are provided in **Extended Methods** (Supplementary).

### Animal studies

#### CanSel screening

To select the cancer cell type for screening, B16-F10 melanoma and LLC lung adenocarcinoma cells, were injected in C57BL/6 mice, whereas 4T1 breast cancer cells in Balb/c mice. For the screening, each AAV9 pool was injected bilaterally in the *tibialis anterior* muscle, followed by LLC injection. Total muscle DNA was extracted after 10 days for barcode amplification and NGS to identify relative barcode (transgene) abundance. To compare different pools, we first calculated the mean log value of the frequency of each transgene in muscles injected with each AAV pool (*mean log control AAV*). Then, we assessed the log of the frequencies of the same transgene in every muscle injected with the same AAV pool and cancer cells (*log AAV LLC*) and normalized the relative abundance of each transgene, as *log AAV LLC: mean log control AAV ratio*. Screening results were validated using LLC or LG1233 (LG) cells [12]. Mice were sacrificed at 10 days.

#### Orthotopic model of lung adenocarcinoma

Adult C57BL/6 mice were injected with either AAV6.2FF-EMID2 or AAV6.2FF-CTR into the left lung parenchyma. Two weeks later, LG cells were injected in the tail vein of the same animals.

#### Orthotopic model of pancreatic adenocarcinoma

Pancreatic ductal adenocarcinoma cells were derived from KPC mice and dissociated into single cells. They were implanted orthotopically in C57BL/6J together with AAV9-EMID2 or AAV9-CTR and sacrificed 4 weeks later.

#### Genetic model of pancreatic adenocarcinoma

To generate KC mice, *LSL-Kras*^*G12D*^ mice were bred with *Pdx1-Cre*. Genotypes were confirmed by PCR amplification of ear snips as previously described [13]. Mice were injected with AAV9-EMID2 and AAV9-CTR at 6 weeks of age and then treated with cerulein to induce acute pancreatitis and trigger pancreatic carcinogenesis [13]. Mice were sacrificed 4 weeks after cerulein injection.

### AAV vectors and next-generation sequencing

The cDNAs corresponding to the mouse secretome library, containing DNA barcode to facilitate identification by Next Generation Sequencing (NGS), were individually cloned into a modified pZac2.1 vector (pGi), and used to produce the corresponding AAV9 vectors. The viral preparations had titers higher than 10^12^ viral genomes/mL [3]. DNA barcode (10–base pair sequence) was specifically amplified and amplicons were sequenced using HighSeq 2000 Illumina, as described and validated [5].

### Primary fibroblast culture

Primary fibroblasts were isolated from the abdominal skin of C57BL/6 and COLL-EGFP/αSMA-RFP [14], digested by an enzyme mixture (collagenase I and DNase II) and plated for 2 h, followed by removal of non-adherent cells. To produce ECM, they were cultured for 9 days on glass coverslips, pre-treated with 0.2% gelatine, 1% glutaraldehyde and 1 M ethanolamine, in a medium supplemented with ascorbic acid and recombinant mouse EMID2 (rEMID2). After ECM decellularization [15], LG cells and NIH-3T3 fibroblasts were seeded on the top.

### Immunofluorescence and image analysis

Cells were fixed with 4% PFA, whereas tissues were either snap-frozen or embedded in paraffin. Cells or tissue were stained using primary and corresponding secondary antibodies, as detailed in **Extended Methods**. Images were acquired using either fluorescent microscope or a confocal microscope. At least 6 images per sample were acquired and analysed using ImageJ2 (Fiji) software.

### Bioinformatic and statistical analysis

The survival analysis was performed using TGCA survival tool [16]. Statistical analyses were performed using GraphPad Prism 7.0 on data expressed as mean ± standard error. Statistical significance was determined using unpaired Student’s T-test and one-way ANOVA with Student-Newman-Keuls correction. A P value lower than 0.05 was considered significant.

## Results

### Optimization of the CanSel strategy

To optimize the model for in vivo selection, we compared the growth pattern of three cancer cell lines (B16-F10 melanoma, 4T1 breast cancer, and Lewis Lung Carcinoma, LLC) upon implantation into syngeneic skeletal muscle, which is highly permissive to AAV transduction [17, 18]. B16-F10 and 4T1 cells formed a compact tumor mass, squishing, but not destroying, surrounding muscle fibers. In contrast, LLC cells exhibited an infiltrative pattern of growth, resulting in the progressive replacement of muscle fibers (Supplementary Fig. S1A). This progressive disappearance of transduced fibers would allow the selection of fibers expressing anti-invasive proteins and thereby resisting to cancer invasion. Consequently, the viral genomes encoding for these anti-invasive proteins were expected to be progressively enriched over time. Thus, the entire CanSel screening was performed using LLC cells. To optimize the timing to retrieve viral genomes, we generated a pilot pool composed of 50 randomly chosen AAV vectors, including the positive control Semaphorin3A (Sema3A), which is known to inhibit cell invasion and tumor growth in vivo [19, 20]. This pool was injected in the *tibialis anterior* muscle of five animals and, after seven days, a time sufficient to drive robust transgene expression by AAV vectors [21], three animals were injected into the same muscles with LLC cells and sacrificed after additional two or ten days. As expected, LLC cells progressively replaced muscle fibers, each one containing a few AAV genomes (Supplementary Fig. S1B, C). As described above, any transgene inhibiting cancer cell invasion, thereby promoting fiber survival, was expected to be enriched. We evaluated barcode frequency, representing the frequency of each transgene in muscles injected with the AAV pool, either in the presence or in the absence of cancer cells, and expressed it as a ratio (Supplementary Fig. S1D). At day ten, when cancer cells have invaded a massive portion of the injected muscles, five factors resulted enriched, including our positive control, Sema3A (Supplementary Table S1). Thus, this time point was chosen for the eventual CanSel experiment, using the whole AAV library.

### In vivo selection identifies secreted proteins inhibiting cancer cell invasion

**Fig. 1:**
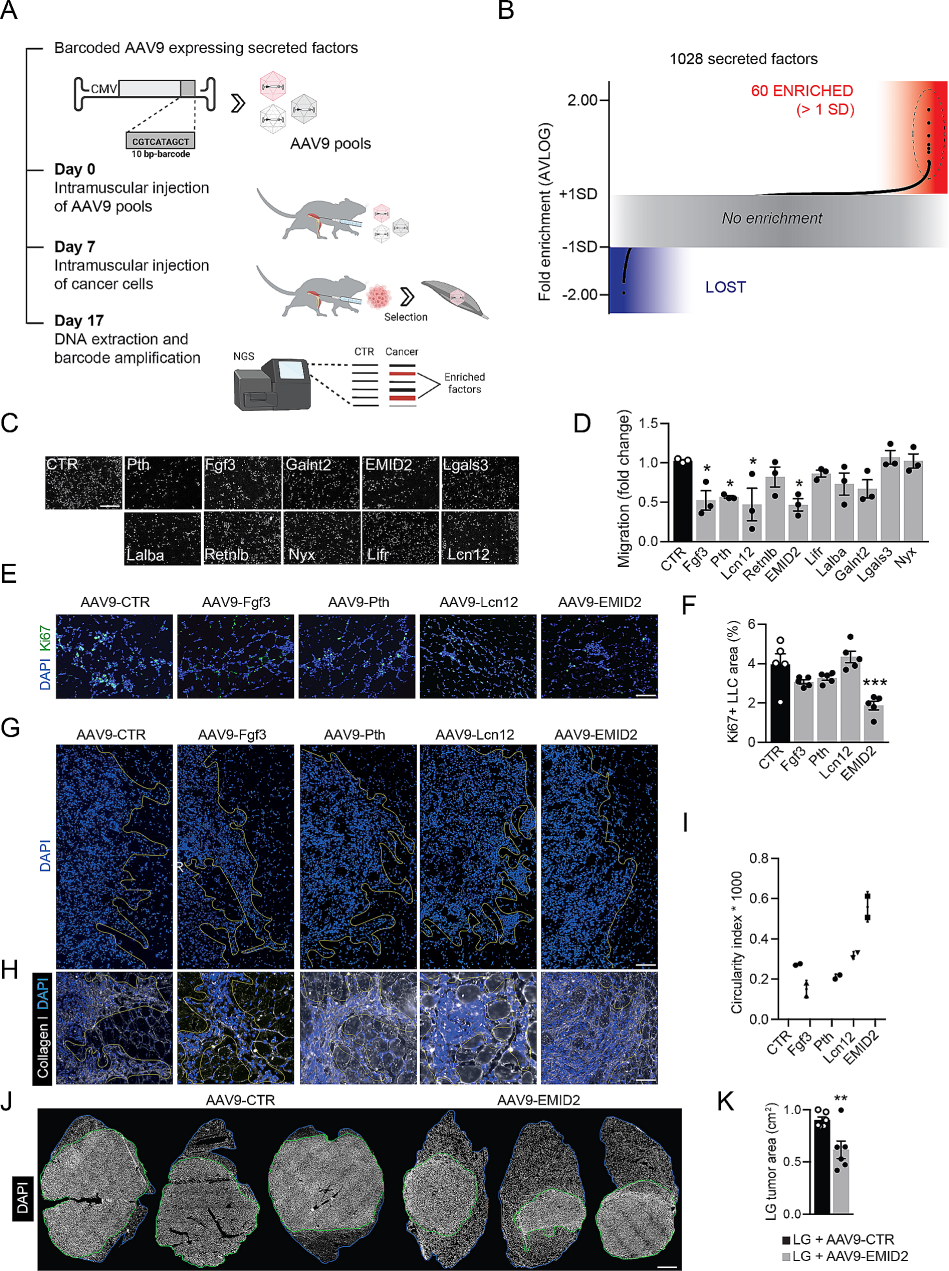
CanSel identifies EMID2 as the most potent factor inhibiting cancer cell invasiveness. **A**. Outline of the CanSel procedure. **B**. Results of the competitive screening ranking 1028 secreted factors. Each dot shows the relative abundance of each transgene in muscles that were injected with both AAV and cancer cells over muscles injected with AAV only. The grey area covers factors that were not enriched. The red area contains factors that were positively selected (enrichment > 1 SD). **C**. Representative images of migrated LLC cells in response to FBS in combination with the indicated factors. **D**. Quantification of LLC migration. **E**. Representative images of LLC cancers, positive for the proliferation marker Ki67, growing in muscles injected with AAV9 vectors expressing the indicated factors. **F**. Percentage of the area covered by Ki67^+^ LLC cells. **G**. Representative images of LG tumor borders with front of invasion indicated in yellow (n = 2 muscles/group). **H**. Higher magnification of peripheral branches showing LG cells migrating on collagen fibers. **I**. Quantification of tumor circularity, a quantitative shape factor of roundness (range, 0–1; 1 = a true circle). **J**. Representative images of LG tumors in muscles injected with AAV9-CTR or AAV9-EMID2. **K**. Quantification of LG tumor size. Scale bar in **C**, **E**, **G** 50 μm, in **H** 100 μm, in **J** 2 mm. In **D**, **F**, **K** data are shown as mean ± SEM and were analyzed by one-way ANOVA with Student-Newman-Keuls correction. *p < 0.05, **p < 0.01, ***p < 0.001

We prepared 21 pools of AAV9, each one composed of an equimolar amount of 50 vectors, carrying transgenes of comparable lengths, as validated by our previous studies [3]. Each AAV pool was injected bilaterally into the *tibialis anterior* muscle. After seven days, three muscles per pool were injected with 5 × 10^4^ LLC cells and harvested after ten additional days for DNA extraction and identification of the relative abundance of each barcode by NGS, as schematically represented in Fig. 1A.

Cumulative barcode recovery was > 70% for 18 out of 21 pools (Supplementary Fig. S2A). The relative abundance of each transgene in its pool is reported in Supplementary Table S2. We first analyzed pools individually and calculated the relative abundance of each transgene in its pool (Supplementary Table S2 and Supplementary Fig. S2B). All pools were eventually ranked together. As shown in Fig. 1B, some transgenes were lost, meaning that they favored cancer cell invasion (blue area), most transgenes were neither lost nor enriched, consistent with their neutral role on cell invasiveness (gray area), and 60 transgenes were enriched (*avlog* > 1SD, red area). The 10 top enriched transgenes were selected for further validation and included parathormone (PTH), Fibroblast Growth Factor 3 (FGF3), polypeptide N-acetylgalactosaminyltransferase 2 (GALNT2), EMID2, galectin 3 (LGALS3), lactalbumin alpha (LALBA), resistin-like beta (RTNLB), nyctalopin (NYX), Leukemia Inhibitory Factor Receptor (LIFR), and lipocalin 12 (LCN12).

Gene ontology analysis revealed that the most abundant categories among both enriched and lost factors were related to ECM remodeling, immunity, angiogenesis and cell migration, confirming our initial aim of selecting factors able to modulate ECM and extracellular space (Supplementary Fig. S3A, B and Supplementary Table S3). Next, we used a transwell invasion assay to further score the top 10 factors, using both LLC cells (Fig. 1C, D) and immortalized mouse aortic endothelial cells (iMAEC), as an additional cell type that populate most cancer types (Supplementary Fig. S3C, D). In either case, the same factors (PTH, EMID2, FGF3, and LCN12) significantly inhibited cell migration. Thus, we selected these four factors for further in vivo validation.

### EMID2 is the most effective protein in inhibiting cancer cell invasiveness in vivo

We produced AAV9 vectors for the individual expression of PTH, EMID2, FGF3, and LCN12 and injected them intramuscularly prior to implantation of LLC cells. As shown in Fig. 1E, F EMID2 was the most potent factor in reducing muscle infiltration by cancer cells, identified by their positivity for the proliferation marker Ki67. We repeated the experiment, using LG cells, which form more compact masses (Supplementary Fig. S1), for easier quantification of tumor size. By doing this experiment, we observed that LG cells expanded with a branching pattern of infiltration (Fig. 1G). At higher magnification, the invasive front was characterized by branches composed of cancer cells growing along collagen extensions and forming road-like structures, radiating perpendicular from the tumor border toward surrounding tissues (Fig. 1H). This pattern was unaffected or even exacerbated by the presence of PTH, FGF3, and LCN12 (Fig. 1G, I). In contrast, EMID2 overexpression resulted in a more circular tumor mass, suggesting reduced invasiveness of cancer cells (Fig. 1G, I). This was further validated in a larger cohort of animals, in which we could detect a significant reduction in tumor growth by EMID2 (Fig. 1J, K).

### Inhibition of TGFβ maturation by EMID2 hampers CAF activation

**Fig. 2:**
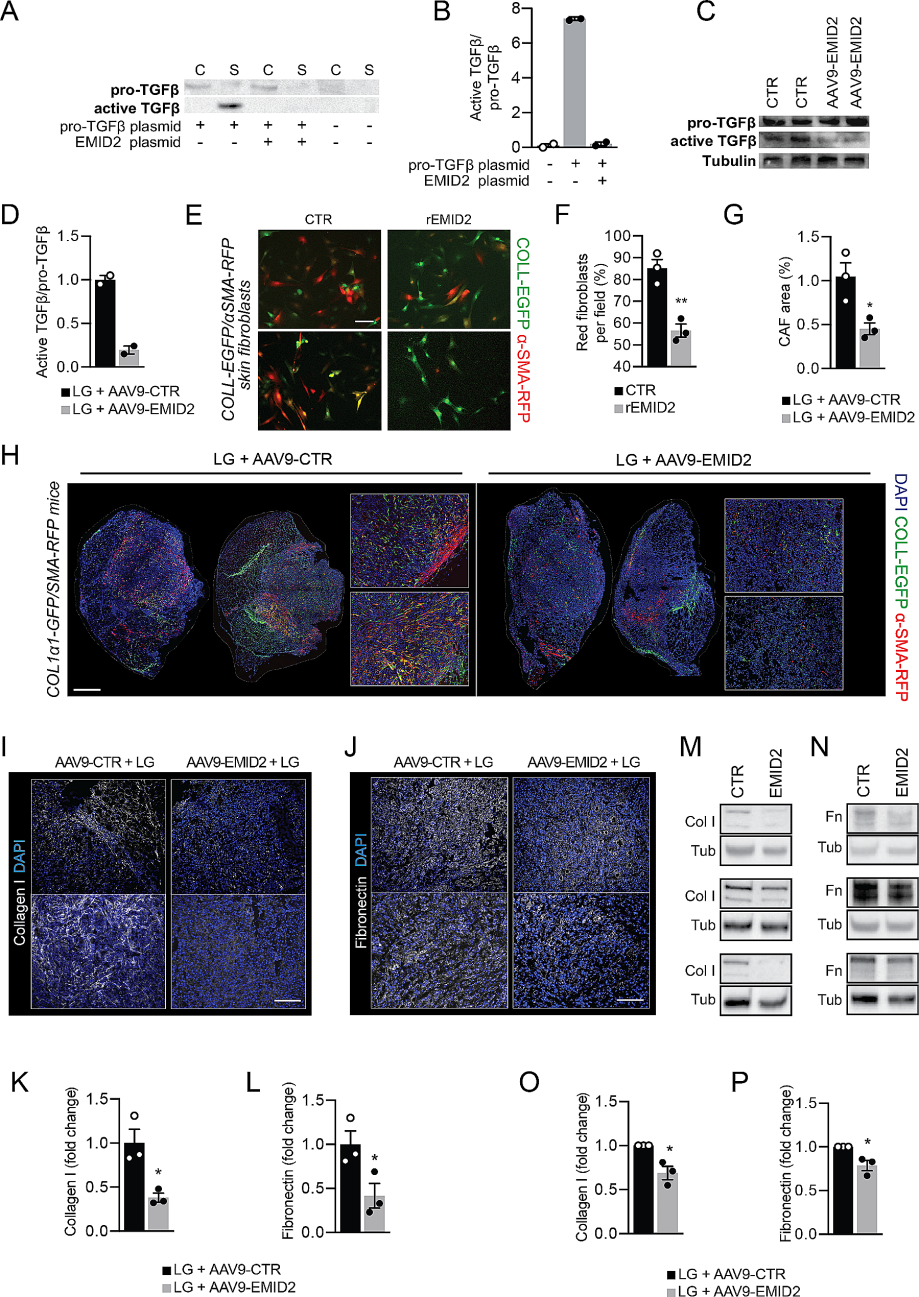
EMID2 inhibits CAF activation and normalizes cancer ECM. **A** Western blot of total cell lysates and supernatants of HEK293T cells transfected with the indicated plasmids. **B** Quantification of active-TGFβ, relative to pro-TGFβ. **C** Western blot of whole muscle tissue injected with LG cells and the indicated vectors. **D** Quantification of active-TGFβ, relative to pro-TGFβ. Tubulin was used as a loading control. **E** Primary fibroblasts from COLL-EGFP/αSMA-RFP mice cultured in absence or presence of rEMID2. **F** Quantification of the percentage of red activated fibroblasts. **G** Quantification of the area covered by EGFP+/α-SMA^+^ CAFs in muscles co-injected with LG cells and AAV9-CTR or AAV9-EMID2. **H** Representative images of muscles co-injected with LG cells and AAV9-CTR or AAV9-EMID2. **I** Representative images of LG tumors in muscles injected with AAV9-CTR and AAV9-EMID2 stained for collagen I. **J** Representative images of LG tumors in muscles injected with AAV9-CTR and AAV9-EMID2 stained for fibronectin. **K.** Quantification of collagen I^+^ area as in H. **L.** Quantification of fibronectin^+^ area as in J. **M.** Western blots showing collagen I (Col I) content in muscles co-injected with LG cells and AAV9-CTR or AAV9-EMID2. **N.** Western blots showing fibronectin (Fn) content in muscles co-injected with LG cells and AAV9-CTR or AAV9-EMID2. Tubulin (Tub) was used as loading control. **O.** Quantification of collagen I as in M. **P.** Quantification of fibronectin as in N. Scale bar in **E** 50 μm, in **H** 1 mm, in **I, J** 100 μm. In **B**, **D**, **F**, **G, K, L, O, P** data are shown as mean ± SEM and were analyzed by one-way ANOVA with Student-Newman-Keuls correction. *p < 0.05, **p < 0.01

To explore the mechanism by which EMID2 inhibits tumor invasion, we analyzed the structural domains of the protein. EMID2 belongs to the EMI domain endowed (EDEN) superfamily of proteins, which all contain a cysteine-rich sequence of approximately 80 amino acids, defined as the EMI domain [22]. This domain is typically found at the N terminus of matricellular proteins that form multimers. Because the EMI domain of Emilin 1 was shown to inhibit TGFβ maturation in the context of hypertension [23], we assessed whether EMID2 could do the same. We transfected HEK293T cells with a plasmid expressing pro-TGFβ, either alone or in combination with a second plasmid expressing EMID2. As shown in Fig. 2A, B, pro-TGFβ was mostly detected inside the cells, while the mature, active form was exclusively present in the cell supernatant. Overexpression of EMID2 did not affect the levels of pro-TGFβ but inhibited its maturation into the active isoform. Next, we wondered whether the same happened in vivo, in EMID2-treated tumors. Consistent with the in vitro data, homogenized EMID2-treated tumors contained lower levels of active TGFβ (shown and quantified in Fig. 2C, D).

Since TGFβ is a key player in stimulating cancer-associated fibroblasts (CAFs), we investigated whether EMID2 could inhibit their activation, using primary skin fibroblasts from COLL-EGFP/αSMA-RFP mice, which simultaneously express the enhanced green fluorescent protein (EGFP) and the red fluorescent protein (RFP) under the control of the collagen α1(I) and the α-smooth muscle actin (αSMA) promoter/enhancer respectively [14]. When kept in culture for five days, these fibroblasts almost completely transdifferentiate into myofibroblasts, resulting in a shift from green to red fluorescence [24].

Administration of rEMID2 significantly reduced the number of RFP^+^ fibroblasts at three days after plating (Fig. 2E, F). The same effect was observed by co-culturing primary COLL-EGFP/αSMA-RFP fibroblasts with LG cells to enhance their differentiation into myofibroblasts (Supplementary Fig. S4A, B). We then assessed whether this inhibition also occurred in tumors in vivo, by injecting LG cells in COLL-EGFP/αSMA-RFP muscles transduced with either AAV9-control or AAV9-EMID2. The area covered by activated CAFs inside tumors was significantly smaller in muscles pre-injected with AAV9-EMID2 compared to those injected with AAV9-control (Fig. 2G, H). The same result was obtained by injecting LG cells in wild type muscles followed by staining with anti-αSMA antibodies (Supplementary Fig. S4C-E). The injection of AAV9-EMID2 in the absence of cancer cells did not induce any change in αSMA expression (Supplementary Fig. S4F).

CAFs are the major source of tumor ECM and a shift from a laminin- to a collagen- and fibronectin-rich environment is known to promote cancer cell invasiveness [25–27]. By analyzing the content of these ECM proteins by immunofluorescence, we observed that both collagen I and fibronectin were reduced by the overexpression of EMID2 (Fig. 2I-L), while laminin was up-regulated to the level of normal muscles (Supplementary Fig. S5A, B). The same data were confirmed by western blot of homogenized tumors implanted in muscles transduced with either AAV9-control or AAV9-EMID2 (Fig. 2M-P).

Thus, EMID2 inhibits TGFβ maturation and, consequently, CAF activation, resulting in a normalized ECM.

### EMID2 reduces matrix stiffness and nuclear YAP localization

**Fig. 3:**
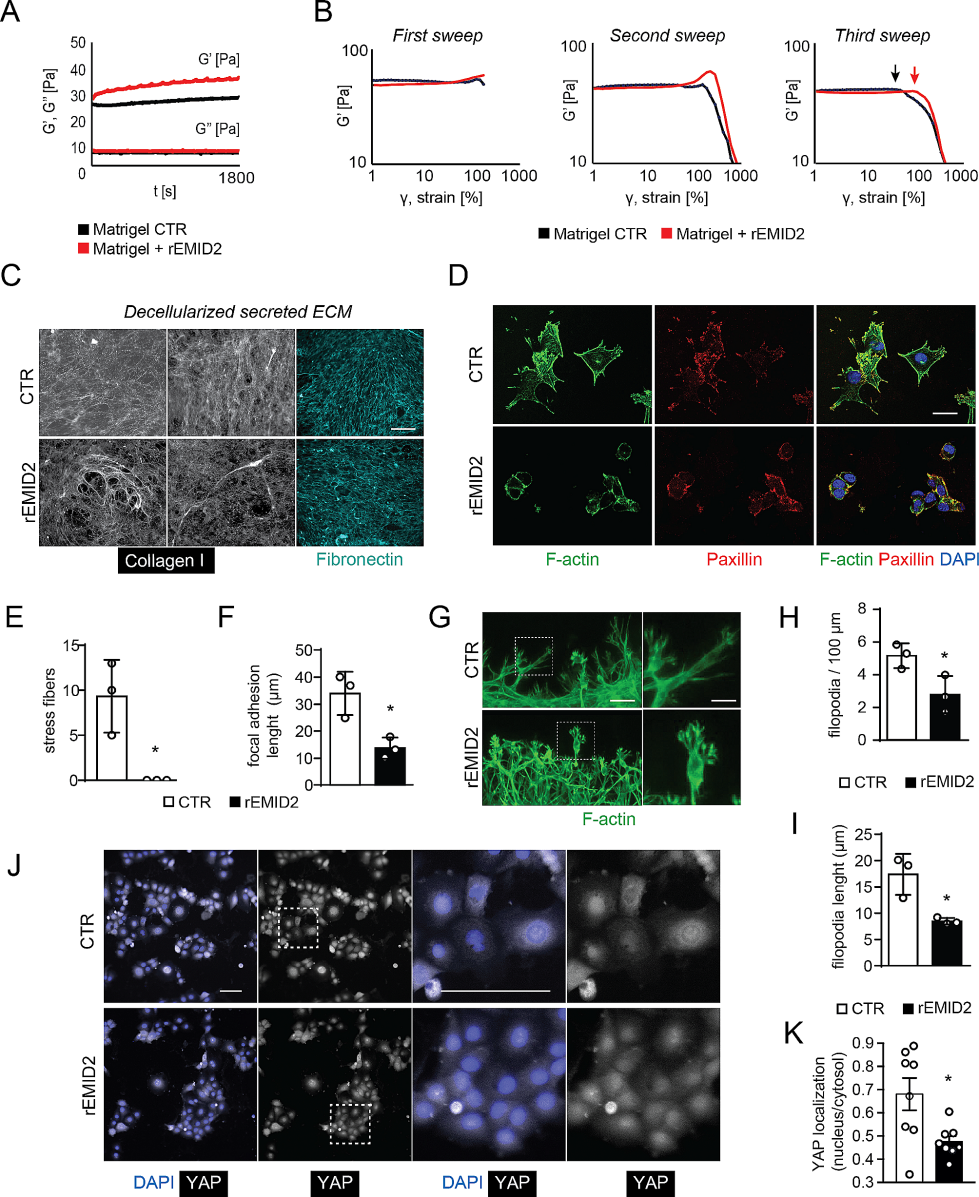
EMID2 incorporation in the extracellular matrix modifies its mechanical properties with consequent cytoskeletal re-organization. **A.** Rheological analysis of Matrigel polymerization in presence of recombinant EMID2 (rEMID2, red) compared to control Matrigel (black). G’, elastic modulus; G’’, viscous modulus. **B.** Strain sweep of Matrigel in the presence of rEMID2 (red) or control (black). Arrows indicate breaking points. **C.** Decellularized ECM secreted by primary fibroblasts cultured in a medium containing rEMID2 or in a control medium. **D.** Representative images of LG cancer cells seeded on Matrigel either in the absence or in the presence of rEMID2. **E.** Quantification of the number of stress fibers per cell, identified by F-actin, in cancer cells seeded on Matrigel either in the absence or in the presence of rEMID2. **F.** Quantification of the length of focal adhesions, identified by paxillin, in cancer cells seeded on Matrigel either in the absence or in the presence of rEMID2. **G.** NIH-3T3 fibroblasts cultured on the decellularized ECM. **H, I.** Quantification of the number and length of filopodia protruding from NIH-3T3 fibroblasts. **J.** Representative images of LG cancer cells seeded on Matrigel either in the absence or in the presence of rEMID2. **K.** Quantification of the ratio of YAP integrated density in the nucleus and cytoplasm of LG cells as in **J**. Scale bar in **C, G, J** 50 μm, in **D** 100 μm. In **E**, **F**, **H**, **I, K**, data are shown as mean ± SEM and were analyzed by Student t-test. *p < 0.05, **p < 0.01

To characterize the effect of EMID2 on ECM mechanical properties, we added recombinant EMID2 to a Matrigel layer and evaluated gelation kinetics of Matrigel with a rheometer. The elastic modulus (G’) and the viscous modulus (G’’) of the samples were measured for 30 min to allow Matrigel polymerization. While the viscous modulus was only minimally increased by the presence of rEMID2 and remained constant during gelation, G’ showed a steep increase during the first minutes and further increased at later times (Fig. 3A). This is consistent with EMID2 interaction with Matrigel proteins and consequent increase in the elastic component of the resulting gel. Next, we applied three subsequent sweeps of increasing strain. By applying a first sweep, resulting in 100% deformation, neither the control Matrigel nor the Matrigel with rEMID2 reached the stiffening limit and their G’ showed a comparable, linear response (Fig. 3B, first panel). This indicates that the gel resisted to the applied forces without breaking. We then applied a second, more intense sweep, resulting in 1000% deformation. The EMID2-containing Matrigel showed a peak in G’, which was not present in the control, which indicates higher resistance (Fig. 3B, second panel). Finally, we applied a third sweep, again resulting in 1000% deformation and acting on the materials previously deformed. The inclusion of EMID2 in the Matrigel again increased resistance, as shown by the higher level of strain at which G’ started to decline (46% in the case of control Matrigel and 92% in the presence of EMID2) (Fig. 3B, third panel). Overall, these rheological measures confirmed that the rEMID2 interacted with Matrigel components and resulted in increased elasticity at high strains. To assess whether EMID2-induced ECM elasticity altered cell adhesion and invasiveness, we cultured primary fibroblasts for nine days, to allow abundant ECM secretion [15], either in the presence or in the absence of rEMID2 (Fig. 3C). We then seeded LG cells on decellularized matrices and stained them with phalloidin and anti-paxillin antibodies to label F-actin cytoskeleton and focal adhesions (FA), which are essential for cell migration [28]. Exposure to EMID2 almost completely abrogated the formation of stress fibers, composed by lamellar F-actin bundles, and reduced the length of FAs, which appeared scattered along the whole cell membrane (Fig. 3D-F). We also seeded NIH-3T3 fibroblasts on the same matrices, as these cells are known to extend multiple and long filopodia prior to their movement [29]. Also in this case, the presence of EMID2 significantly reduced both the number and length of filopodia (Fig. 3G-I).

As changes in ECM mechanical properties and cytoskeletal actin organization are generally transduced by the Hippo pathway [30], we monitored YAP localization in LG cancer cells seeded on control and rEMID2-contaning matrices and found that YAP was preferentially excluded from the nucleus in the presence of rEMID2 (Fig. 3J, K), paralleled with reduced expression of YAP target genes involved in cancer cell migration, invasiveness and metastasis (Supplementary Fig. S6).

Thus, EMID2 significantly increases ECM elasticity, resulting in reduced cancer cell adhesion, filopodia formation and nuclear YAP localization.

### EMID2 inhibits Tumor growth and dissemination in clinically relevant animal models

We validated the anti-invasive effect of EMID2 in clinically relevant animal models of highly invasive and metastatic cancer types, such as lung and pancreatic cancer [31, 32].

**Fig. 4:**
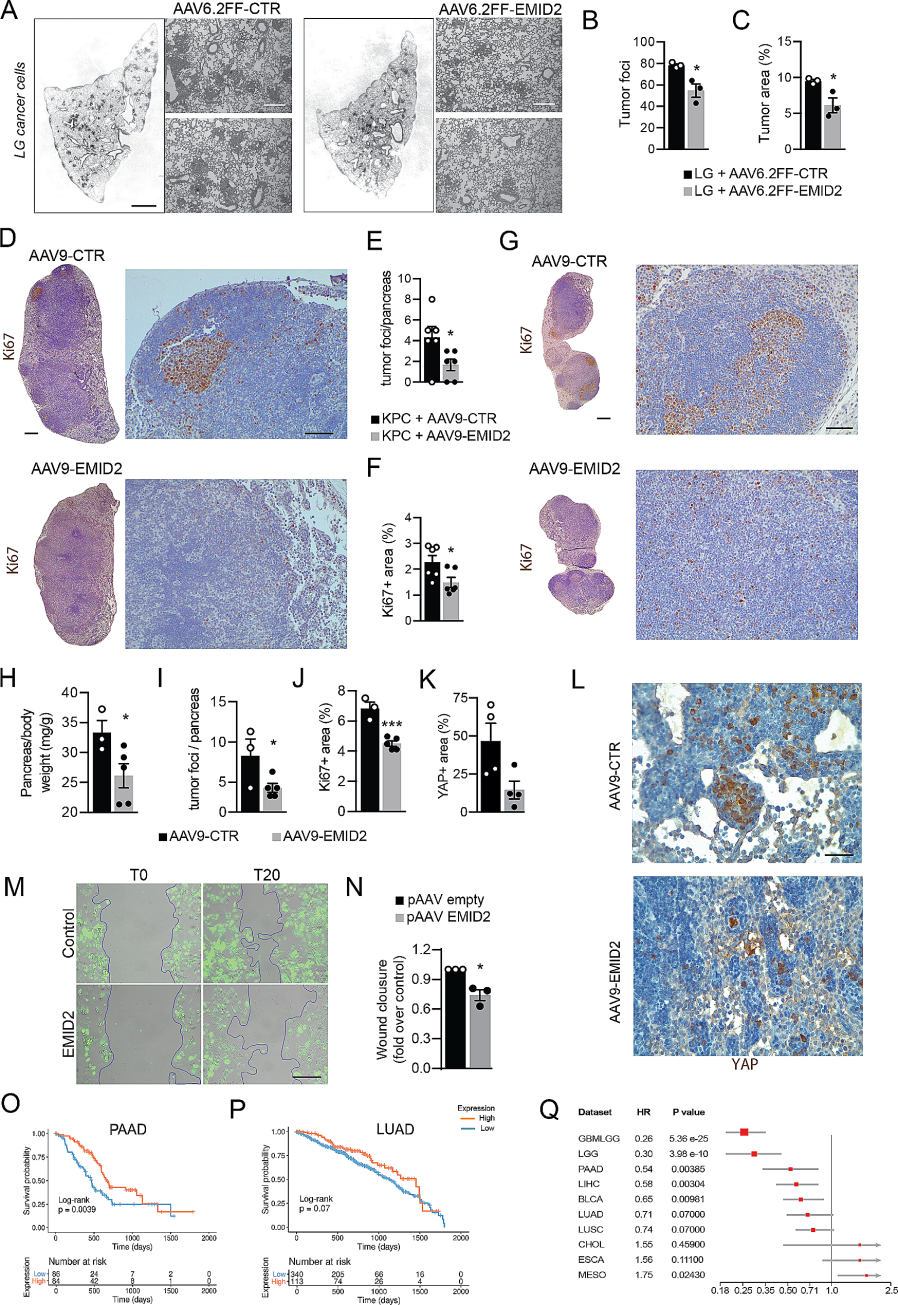
EMID2 overexpression inhibits the growth and dissemination of murine and human cancers. **A** Representative hematoxylin/eosin staining of total lung and zoomed fields of LG tumor mass. **B** Number of LG tumor foci. **C** Area covered by LG cells. **D** Representative images of total pancreas and zoomed fields injected with KPC cells. KPC cells are stained for Ki67. Nuclei are counterstained with hematoxylin. **E** Number of KPC tumor foci per pancreas. **F** Area covered by Ki67^+^ KPC cells. **G** Representative images of total pancreas and zoomed fields of KC mice. Cancer cells are stained for Ki67. Nuclei are counterstained with hematoxylin. **H** Pancreas/body weight for each mouse. **I** Number of tumor foci per pancreas. **J** Area covered by Ki67^+^ cancer cells. **K.** Area covered by YAP^+^ cancer cells on tumor area. **L.** Representative images of the pancreas of KC mice stained for YAP. **M.** Representative images of wound healing assay with EGFP^+^ Panc1 cells co-cultured human fibroblasts overexpressing EMID2 or control fibroblasts, at the indicated time points. **N.** Wound closure at T20 relative to T0. **O, P.** Kaplan-Meier curves of patients affected by pancreatic adenocarcinoma (PAAD) and lung adenocarcinoma (LUAD), according to the expression levels of EMID2, at the indicated time points from diagnosis. **Q.** Forest plot indicating the Hazard Ratio (HR) for the most aggressive human solid cancers. GBMLGG: glioblastoma multiforme and low-grade glioma, LGG: low grade glioma, PAAD: pancreatic adenocarcinoma, LIHC: liver hepatocellular carcinoma, BLCA: bladder urothelial carcinoma, LUAD: lung adenocarcinoma, LUSC: lung squamous cell carcinoma, CHOL: cholangiocarcinoma, ESCA: esophageal carcinoma, MESO: mesothelioma. Square size reflects the weight of the significance. Source: TCGA database. Scale bar in **A** (whole lung), **D, G** (whole pancreas) 1 mm, in **A, D, G** (magnification) 200 μm, in **L**, **M** 50 μm. In **B**, **C**, **E**, **F**, **H, I, J, K, N** data are shown as mean ± SEM and were analyzed by one-way ANOVA with Student-Newman-Keuls correction. *p < 0.05, ***p < 0.001

First, we used an orthotopic model of lung cancer, by systemically injecting LG cells, which spontaneously colonize the lung, forming numerous neoplastic nodules [24]. In this case, we used AAV6.2FF vectors, herein referred to as AAV6, due to the high tropism of this AAV serotype for the lung [33]. Delivery of AAV6-EMID2 significantly increased EMID2 levels compared to endogenous expression (Supplementary Fig. S7A, B), and resulted in reduced number of tumor foci and their total extension (Fig. 4A-C).

Next, we moved to a model of pancreatic ductal adenocarcinoma. KPC cells were orthotopically implanted in the pancreas of C57BL/6 mice, together with AAV9-EMID2 or AAV9-control vectors, due to the tropism of AAV9 for the pancreas [34]. We assessed KPC proliferation by immunostaining for Ki67 and found that the number of proliferating cancer cells, as well as the tumor size, were significantly reduced upon EMID2 overexpression in the pancreas (Fig. 4D-F). In addition, the delivery of AAV9-EMID2 to the pancreas inhibited cancer cell dissemination to the lung, resulting in decreased density of Ki67^+^ cancer cells in EMID2-treated animals compared to controls (Supplementary Fig. 8A, B).

Finally, we used a genetic model of pancreatic cancer in KC mice [13]. Also in this case, overexpression of EMID2 resulted in a significant reduction in overall pancreatic weight, paralleled by fewer Ki67^+^ cancer cells and primary tumor nodules (Fig. 4G-J). Consistent with our data on LG cells, overexpression of EMID2 resulted in reduced levels of YAP in pancreatic cancers (Fig. 4K, L). Even more evident was the effect on lung cancer dissemination, with a minimal number of metastatic, Ki67^+^ cancer cells detectable in the lungs of EMID2-treated animals (Supplementary Fig. 8C, D).

### EMID2 is a positive prognostic marker in aggressive human cancer

To verify whether EMID2 also inhibited the migration of human pancreatic cancer cells, we transfected human fibroblasts with a plasmid encoding for EMID2 and, after two days, added Panc1 cells, previously labeled with a fluorescent membrane dye. As shown in the scratch assay in Fig. 4M, N, EMID2 overexpression significantly inhibited Panc1 cell migration.

To further explore the relevance of EMID2 in human cancer, we interrogated The Cancer Genome Atlas (TCGA) to analyze the correlation between EMID2 expression levels and survival rates in multiple cohorts of patients diagnosed with aggressive cancer (https://www.nuffieldtrust.org.uk/resource/cancer-survival-rates). First, we focused on pancreatic and lung adenocarcinoma (PAAD and LUAD), to validate the protective effect of EMID2 observed in animal models. As shown in Fig. 4O, P, Kaplan-Meier curves show increased survival in patients with high expression of EMID2 in their tumor. Next, we extended our analysis to lung squamous cell carcinoma (LUASC), mesothelioma (MESO), glioblastoma and glioma (GBMLGG, LGG), liver hepatocarcinoma (LIHC), cholangiocarcinoma (CHOL), bladder cancer (BLCA), and esophageal cancer (ESCA). As shown in the forest plot of Fig. 4Q, the hazard ratio (HR) value for most of these tumors was < 1, indicating that high expression of EMID2 was associated with increased probability of survival. This association was significant for GBMLGG, LGG, PAAD, LIHC and BLCA (p-value < 0.05). The size of each square in Fig. 4Q reflects the weight of the significance, which was highest for glioma and glioblastoma. The protective effect of high EMID2 expression was most evident for GBMLGG, LGG, LIHC and BLCA, as shown in Supplementary Fig. S9.

Thus, high expression levels of EMID2 are associated with a better prognosis in most aggressive human cancers.

## Discussion

Existing cancer therapies fail to hamper tumor cell invasiveness. Here we took advantage of CanSel, an innovative in vivo selection strategy [3], to identify the most potent proteins inhibiting cancer cell invasiveness, tumor growth and dissemination.

The top-ranked protein identified by CanSel was EMID2, which has the potential to make a significant impact in cancer therapy, as (i) it reduces tumor growth and dissemination in in vivo models of lung and pancreatic cancer, (ii) it modifies the composition of cancer ECM, reducing cell invasiveness, and (iii) its high expression levels are associated with good prognosis in aggressive human cancers.

The power of CanSel in identifying and ranking factors with anti-invasive properties relies on the screening of thousands of molecules in a totally unbiased manner, in the absence of any prior knowledge on their function. Gene ontology analysis on both enriched and lost transgenes indicated that out of > 1000 transgenes included in the AAV library, the top 60 encoded mainly for proteins associated with ECM (fibulins, galectins and collagens), angiogenesis (members of the Vascular Endothelial Growth Factor family) and immune response (interleukins, chemokines). These processes all pertain to extracellular milieu, which is consistently altered in aggressive cancers and, therefore, it represents an attractive target for therapy [35].

EMID2 stood out as the most potent factor in both inhibiting cell invasiveness in vitro and reducing tumor growth and dissemination in vivo. After initial comparison of the 10 top hits, we selected the best performing 4 proteins for in vivo validation. More specifically, we assessed their capacity to modify the shape of the invasive front. While in control tumors, the leading edge contained collagen and fibronectin bundles, perpendicularly aligned with the tumor border, which steers cancer cell escape from the primary tumor [36–38], EMID2-overexpressing tumors had a round border, with few infiltrating branches.

EMID2 belongs to the EDEN superfamily, which includes members sharing an EMI domain [22, 39]. Two additional members of this family, Emilin1 and Emilin2, resulted enriched in their respective AAV9-pool, and scored high in the final ranking (33rd and 77th position, respectively), confirming a relevant role of these ECM proteins in inhibiting tumor invasiveness [40, 41]. While the function of Emilins has been extensively investigated in other fields, scant information is available for EMID2, which confirms the power of CanSel in selecting novel molecules. Two studies have associated *EMID2* to pathological and developmental processes characterized by abnormal ECM, such as asthma, nasal polyps and collagen fibrillogenesis in the cornea [42, 43].

Our data show that EMID2 overexpression normalizes ECM composition, reducing the density of collagen I and fibronectin, which are known to support tumor growth and invasiveness [44, 45]. In advanced tumors, TGFβ stimulates the activation of CAFs, leading to ECM remodeling [46], which in turn primes tumor cells for distal dissemination [47]. Consistent with previous data on Emilin1 [23], we observed a significant reduction of active TGFβ in EMID2-treated cells and tumors, with reduced CAF activation.

ECM stiffness is generally higher in tumor than in normal tissues, which favors cancer cell dissemination and reduces drug and immune cell penetration in multiple cancer types, including breast, liver, lung and pancreatic cancer [48, 49]. EMID2 reduces ECM stiffness both in vitro and in vivo, resulting in reduced nuclear YAP.

Most importantly, overexpression of EMID2 at the site of primary tumors in the pancreas, significantly reduced the metastatic burden in the lung, paving the way to its therapeutic use in disseminated human cancers, in combination with standard therapies. Consistently, high expression of EMID2 is a favorable prognostic factor for most aggressive human cancers. The anti-cancer activity of EMID2 is consistent with high level of methylation of its gene in both LUAD and PAAD cells compared to the corresponding healthy tissues, as detected using the (http://www.bioinfo-zs.com/smartapp/) and the MethMarkerDB! (https://methmarkerdb.hzau.edu.cn) databases.

Our initial idea of using a library of secreted proteins has obvious therapeutic implications. Being a secreted protein, EMID2 could be administered as a recombinant protein, avoiding the hurdles related to the use of viral vectors and exploiting the production platforms developed for other collagenous proteins, already used for human therapy [50–52].

To what extent the therapeutic activity of EMID2 could act synergistically with other treatments (i.e. immunotherapy, integrin inhibitors, anti-angiogenic therapy) remains an outstanding issue, which deserves further investigation.

## Conclusions

This is the first in vivo screening of the whole mouse secretome for the identification of proteins able to inhibit the invasive properties of aggressive cancer cells. EMID2 was selected as the most potent protein, able to modulate cancer ECM and reduce both the growth of aggressive pancreatic and lung cancers, and their dissemination. Being a secreted protein, EMID2 could be delivered as a recombinant protein or mRNA, avoiding the hurdles related to the use of viral vectors.

### Electronic supplementary material

Below is the link to the electronic supplementary material.

**Supplementary Material 1:** **Table S1.** Composition of the pilot AAV9 pool

**Supplementary Material 2:** **Table S2.** Relativa abundance of each transgene in each pool (each pool is in a different sheet)

**Supplementary Material 3:** **Table S3.** Gene ontology of enriched and lost factors

**Supplementary Material 4:** Supplementary figures and methods

## Data Availability

All data supporting the findings of this study will be provided with the manuscript as raw data.

## Abbreviations

AAV: Adeno-associated virus
α-SMA: α-smooth muscle actin
BLCA: Bladder cancer
CAR: Chimeric antigen receptor
CAF: Cancer associated fibroblasts
CHOL: Cholangiocarcinoma
ECM: Extracellular matrix
EDEN: EMI domain endowed
EGFP: Enhanced green fluorescent protein
EMID2: EMI domain-containing protein 2
ESCA: esophageal cancer
FGF 3: Fibroblast Growth Factor 3
GALNT2: N-acetylgalactosaminyltransferase 2
GBMLGG: Glioblastoma
HR: Hazard ratio
iMAEC: Immortalized mouse aortic endothelial cells
LALBA: Lactalbumin alpha
LCN12: Lipocalin 12
LGALS3: galectin 3
LUAD: Lung adenocarcinoma
LUASC: Lung squamous cell carcinoma
LGG: Glioma
LIFR: Leukemia Inhibitory Factor Receptor
LIHC: Liver hepatocarcinoma
LLC: Lewis lung cancer
MESO: Mesothelioma
NGS: Next generation sequencing
NYX: Nyctalopin
PAAD: Pancreatic ductal adenocarcinoma
PTH: Parathormone
RFP: Red fluorescent protein
RTNLB: Resistin-like beta
Sema3A: Semaphorin 3 A
TGF-β: Tumor growth factor-β
YAP: Yes associated protein
