# Supplementary material for: EMID2 is a novel biotherapeutic for aggressive cancers identified by in vivo screening
Source: J Exp Clin Cancer Res. 2024 Jan 10;43:15. doi: 10.1186/s13046-023-02942-4 (PMC10777502; doi:10.1186/s13046-023-02942-4)
Supplement: Supplementary file 1 — Supplementary Material 1: Table S1. Composition of the pilot AAV9 pool [file 13046_2023_2942_MOESM1_ESM.docx]

| **Pilot Pool 50 AAV9** | **Fold enrichment** | |
| --- | --- | --- |
| **Transgene** | **Day2** | **Day 10** |
| **Cp** | **1,38** | **2,55** |
| **Olfml2b** | **1,29** | **2,28** |
| Emilin2 | 0,78 | **1,67** |
| Man2b2 | 1,04 | **1,65** |
| Sema3a | 1,08 | **1,57** |
| Spink5 | 0,79 | 1,44 |
| AK053037 | 0,78 | 1,43 |
| Morc2a | 1,13 | 1,37 |
| Furin | 1,21 | 1,32 |
| **Adamts16** | **1,27** | 1,28 |
| Itih1 | 1,16 | 1,28 |
| Loxl3 | 0,91 | 1,23 |
| Adamtsl2 | 1,05 | 1,20 |
| Hhipl1 | 1,05 | 1,17 |
| **Lepr** | **1,25** | 1,16 |
| Eln | 1,17 | 1,15 |
| **C2** | **1,44** | 1,14 |
| Cfb | 0,96 | 1,14 |
| Ace2 | 1,12 | 1,07 |
| Bcan | 1,10 | 1,07 |
| Heg1 | 1,23 | 1,05 |
| St14 | 1,14 | 1,05 |
| Comp | 0,87 | 1,04 |
| Cpxm2 | 0,96 | 1,03 |
| Sema3f | 1,04 | 1,03 |
| Pcsk1 | 0,91 | 1,01 |
| Postn | 1,00 | 1,01 |
| Dpp4 | 1,06 | 0,99 |
| Emilin1 | 0,81 | 0,99 |
| Plg | 0,91 | 0,99 |
| Sema3c | 0,99 | 0,96 |
| **C6** | **1,38** | 0,95 |
| Fstl5 | 1,17 | 0,95 |
| Spon1 | 1,05 | 0,91 |
| **Adamts15** | **1,57** | 0,90 |
| Matn2 | 0,93 | 0,90 |
| BC046331 | 1,04 | 0,89 |
| Qsox1 | 1,00 | 0,85 |
| Lepre1 | 0,89 | 0,84 |
| Gsn | 0,89 | 0,83 |
| Vwa5a | 0,95 | 0,83 |
| Itih3 | 1,09 | 0,82 |
| Svs1 | 0,93 | 0,82 |
| Prss12 | 0,90 | 0,77 |
| Adamts4 | 1,04 | 0,75 |
| Nell2 | 1,12 | 0,74 |
| **Dag1** | **1,31** | 0,73 |
| Thbs3 | 1,17 | 0,50 |
| Ephb6 | 1,03 | 0,45 |
| Egflam | 1,13 | 0,12 |
