# Supplementary material for: EMID2 is a novel biotherapeutic for aggressive cancers identified by in vivo screening
Source: J Exp Clin Cancer Res. 2024 Jan 10;43:15. doi: 10.1186/s13046-023-02942-4 (PMC10777502; doi:10.1186/s13046-023-02942-4)
Supplement: Supplementary file 4 — Supplementary Material 4: Supplementary figures and methods [file 13046_2023_2942_MOESM4_ESM.pdf]

Supplementary material

SUPPLEMENTARY FIGURES

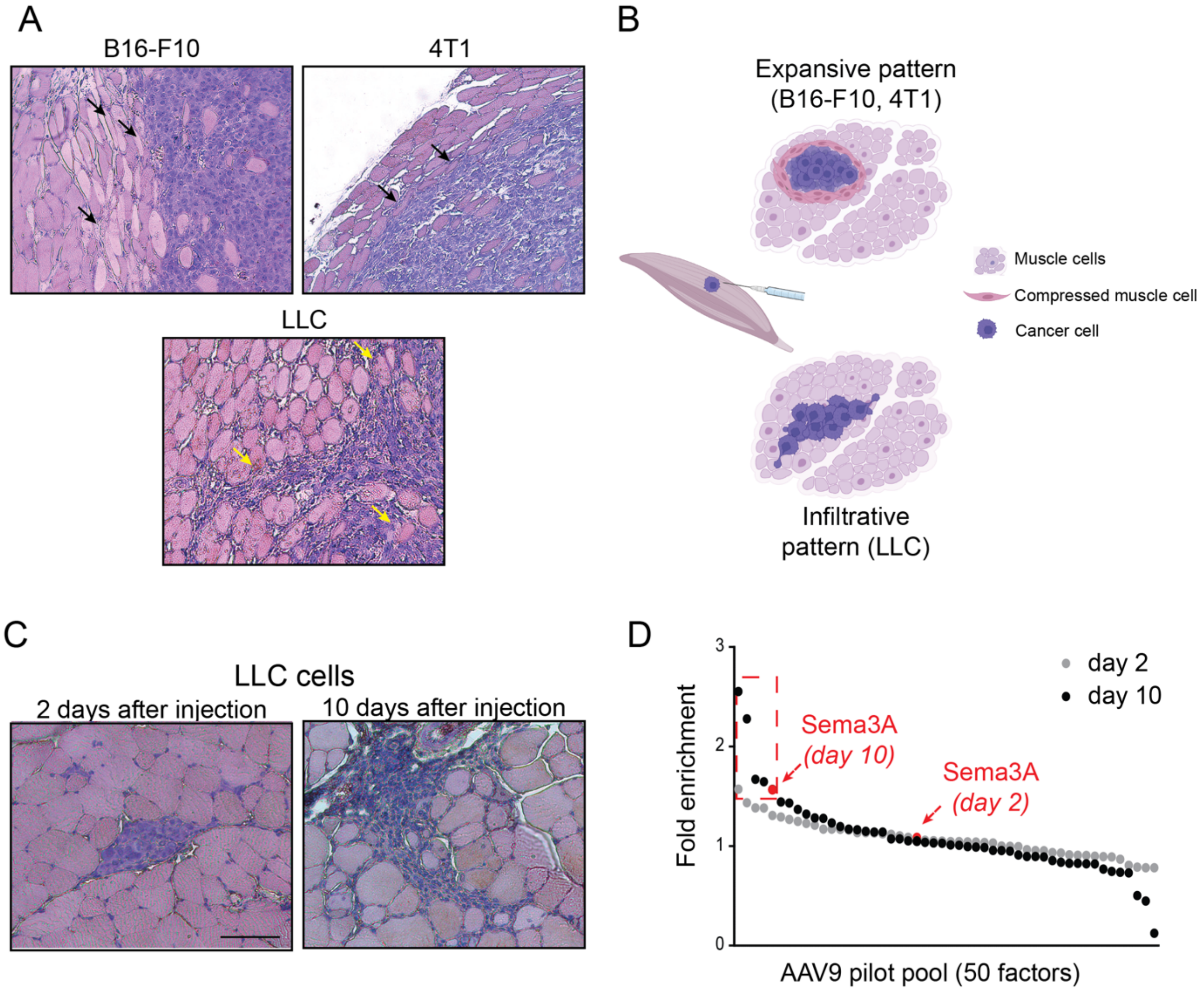

Supplementary Figure S1. A. Hematoxylin and eosin staining of tibialis anterior muscles injected with the indicated cancer cell lines (n = 3 muscles/group). Black arrows show fibers compressed by B16-F10 and 4T1 cells, which expanded without invading adjacent tissues. Yellow arrows show muscle fibers progressively replaced by infiltrating LLC cells. Scale bar, 50 μm. **B**. Schematic representation of the expansive and infiltrative pattern of growth of B16-F10, 4T1 and LLC cells, respectively. Only LLC cells are expected to progressively replace transduced muscle fibers, thus exerting the selective pressure required for the selection of transgenes inhibiting cancer invasiveness. **C.** Representative images of LLC cells at two and ten days after intramuscular injection (n=3 muscles/group). Scale bar, 50 μm. **D**. Results of a pilot experiment in which an AAV9 pool composed of 50 secreted factors was injected in the tibialis anterior muscle, followed by LLC cells. The graph shows the enrichment of each transgene at either two (grey dots) or ten (black dots) days after LLC cell injection. Red dots indicate Semaphorin3A (Sema3A), used as a positive control.

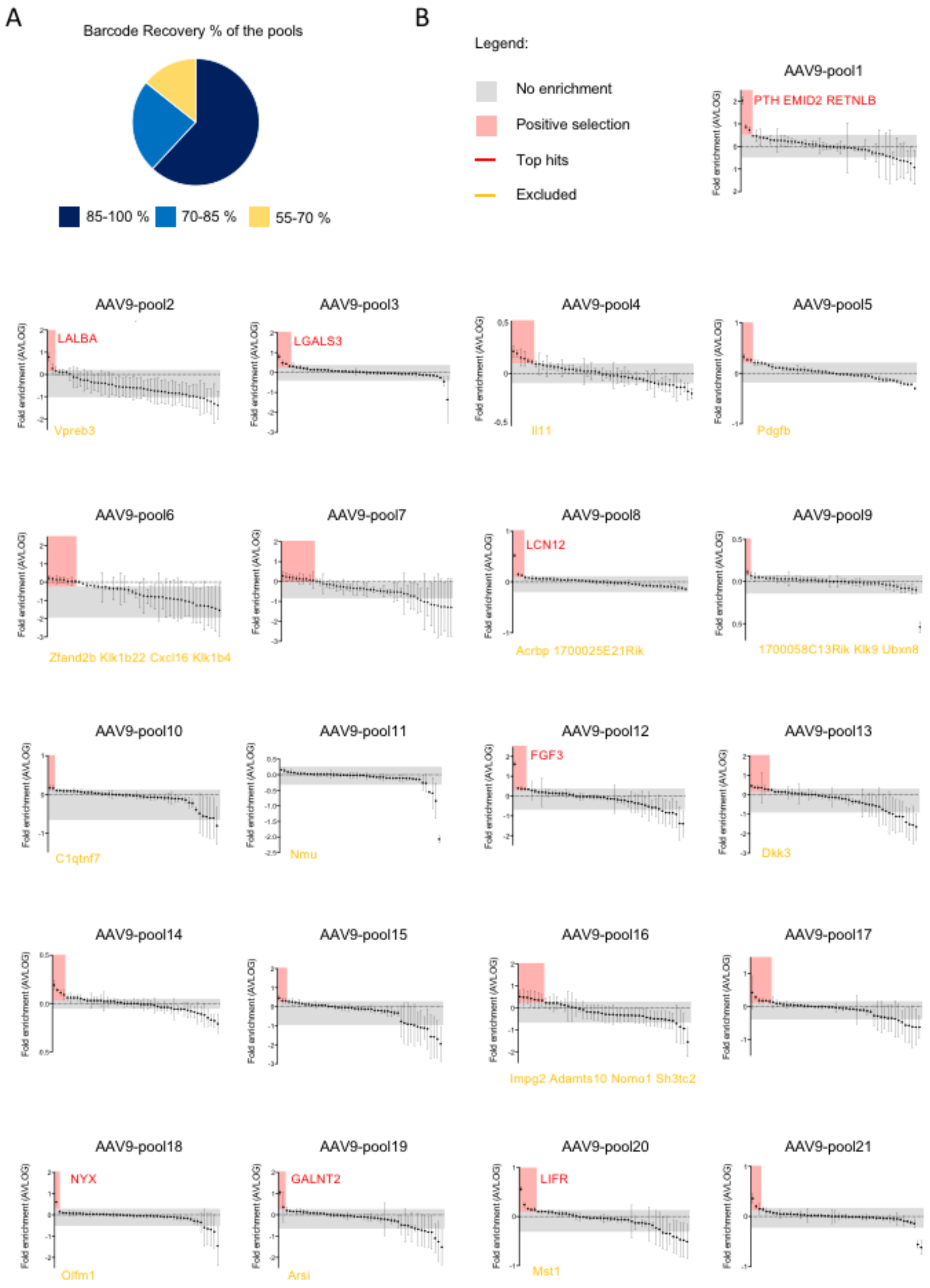

Supplementary Figure S2. A. Pie chart showing the percentage of barcode

recovery for the 21 pools. **B**. Graphs showing the enrichment of each transgene for the individual 21 AAV9 pools. Each dot shows the relative abundance of each transgene in muscles injected with both AAV and LLC cells over muscles transduced with AAV only. The grey area includes transgenes that were not enriched. The red area highlights transgenes that were positively selected (enrichment > 1 SD). Top hits, identified by merging all pools as indicated in Figure 1, are indicated in red. Transgenes that were not detected either in the original viral lysate or in transduced muscles are indicated in orange under each graph. Data are shown as mean ± SEM.

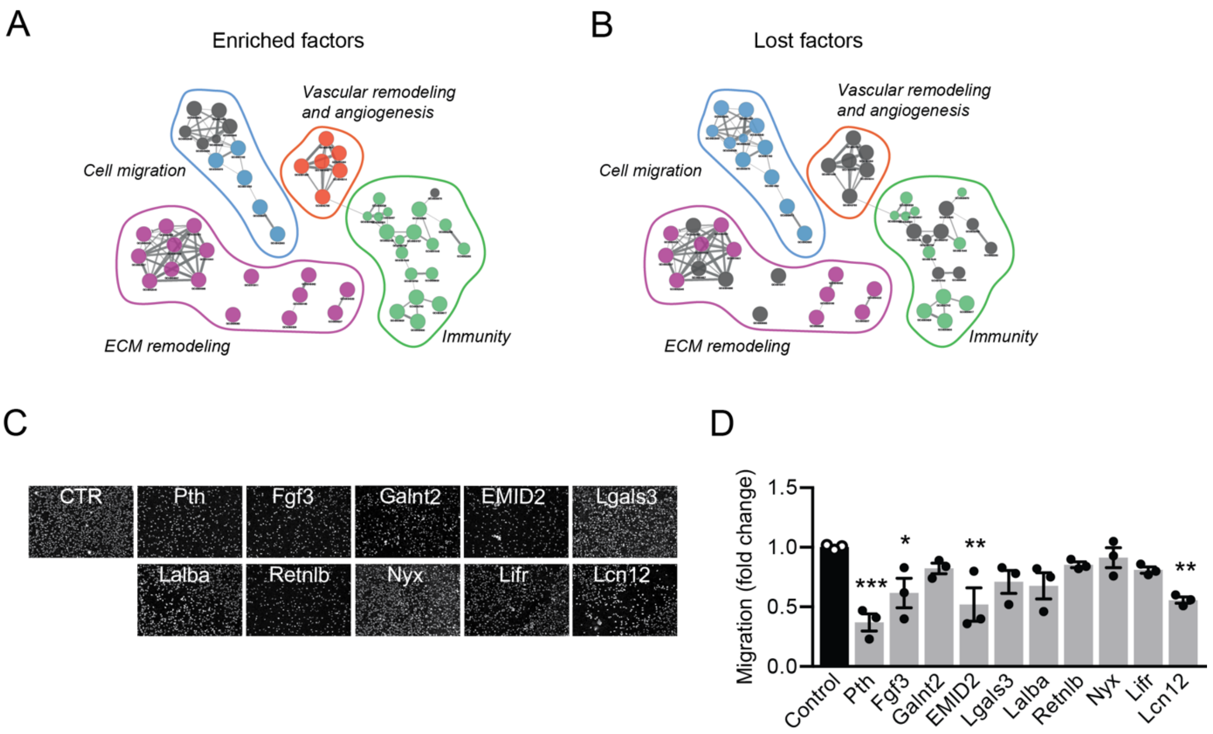

Supplementary Figure S3. A, B. To highlight biological pathways associated with enriched (A) and lost (B) factors, a network of the most significant ($p < 0.05$) Gene Ontology biological processes was drawn using the 'ClueGO' Cytoscape plug-in. Node color (purple, green, red and blue) indicates a significant association of a pathway with factors, while the node size denotes the number of genes involved in the process. The edge thickness depicts term-term interaction, in terms of Cohen's kappa coefficient: the thicker the link, the higher the number of genes shared by terms. **C**. Representative images of migrated iMAECs in combination with the indicated factors. **D**. Quantification of iMAEC migration. Data are shown as mean ± SEM and were analyzed by one-way ANOVA with Student-Newman-Keuls correction. *$p < 0.05$, **$p < 0.01$, ***$p < 0.001$.

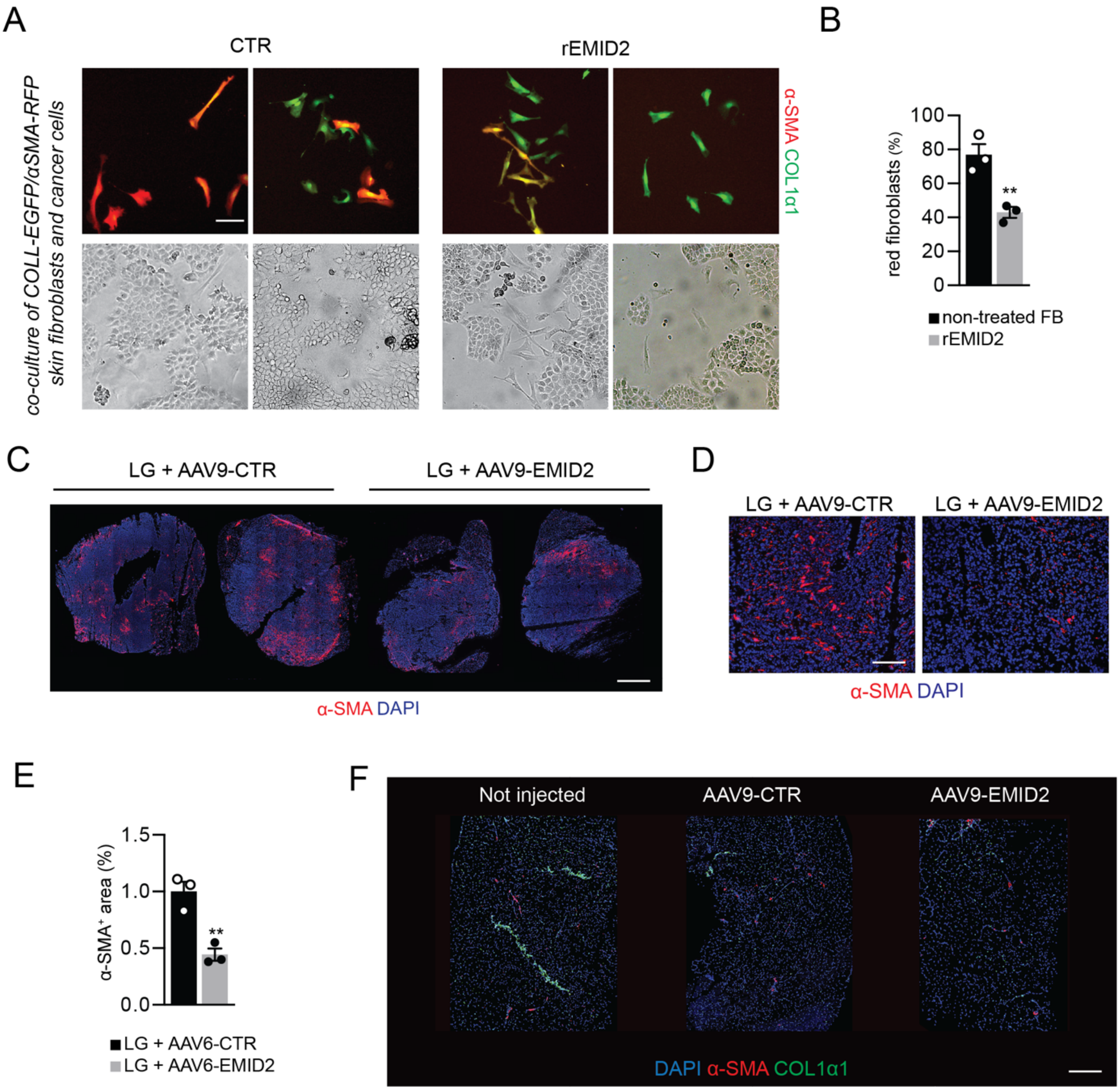

Supplementary Figure S4. A. Primary fibroblasts from COLL-EGFP/αSMA-RFP mice co-cultured with LG cancer cells, in either absence or presence of rEMID2. Scale bar, 50 µm. **B**. Quantification of red fibroblasts. **C.** Representative whole sections of muscles co-injected with LG cells and AAV9-CTR or AAV9-EMID2, stained using α-SMA antibody. Scale bar, 0,2 cm. **D.** High magnification images of sections in C to better visualize α-SMA^{+} CAFs. Scale bar, 50 µm. **E.** Quantification of the area covered by α-SMA^{+} CAFs in muscles co-injected with LG cells and AAV9-CTR or AAV9-EMID2 (n = 3 muscles/group). **F.** Representative images of the whole tibialis anterior muscle of COLL-EGFP/αSMA-RFP mice, that were injected with AAV9-CTR or AAV9-EMID2, or not injected. Scale bar, 0,2 cm. Data in B and E are shown as mean ± SEM, and were analysed by unpaired Student's T-test. $^{**}p < 0.01$.

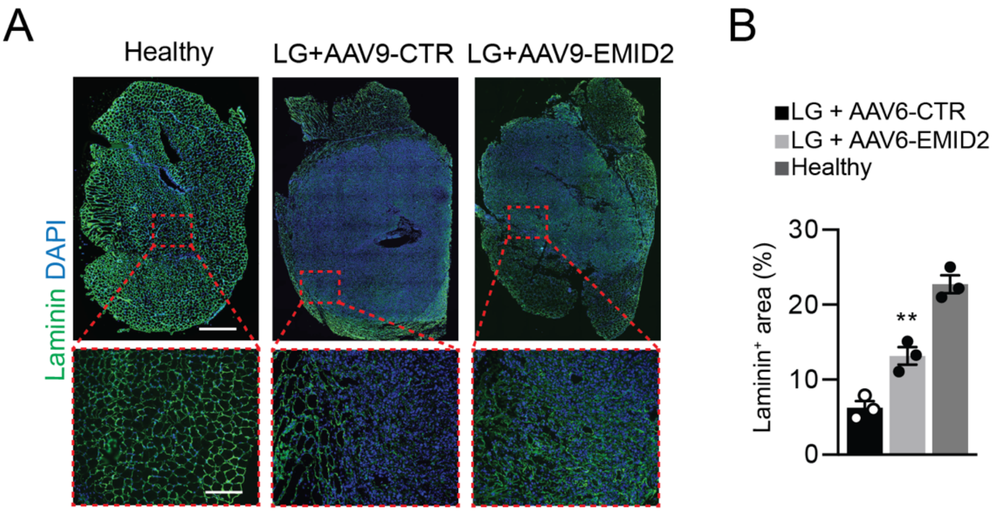

Supplementary Figure S5. A. Representative images of laminin staining of healthy muscle and LG tumors in muscles injected with AAV9-CTR or AAV9-EMID2 (n = 3 muscles/group]. Scale bar, 0,2 cm in the upper panels and 50 µm in the lower panels. **B.** Quantification of laminin$^+$ pixels in the tumor area in the total muscle section. Data are shown as mean ± SEM, and were analysed by one-way ANOVA with Student-Newman-Keuls correction. **p < 0.01.

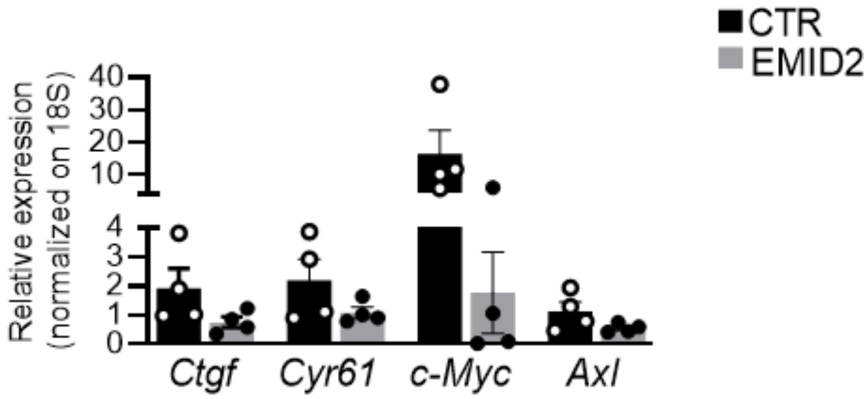

Supplementary Figure S6. Quantification of the expression level of the indicated YAP target genes relative to the housekeeping gene 18S. Data are shown as mean ± SEM and were analyzed by one-way ANOVA with Student-Newman-Keuls correction.

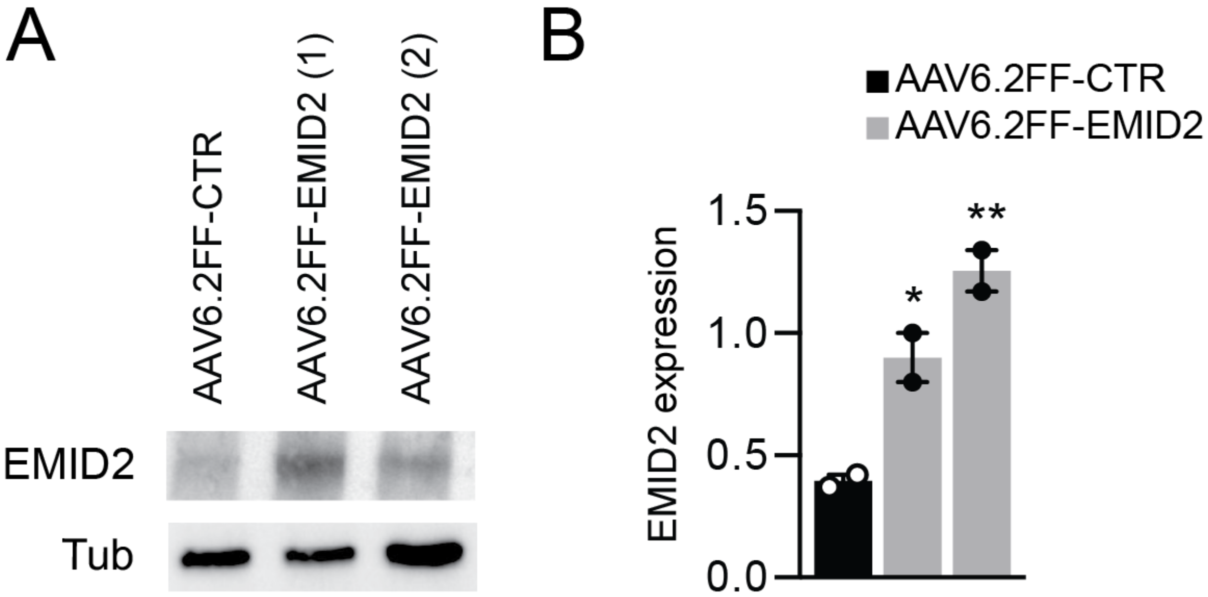

Supplementary Figure S7. A. Western blot of lung lysates transduced with either AAV6.2FF-CTR or AAV6.2FF-EMID2 for EMID2 and tubulin (Tub), used as a loading control. **B.** Quantification of EMID2 protein level in the lung, relative to tubulin. Data are shown as mean ± SEM and were analyzed by one-way ANOVA with Student-Newman-Keuls correction. *$p < 0.05$, **$p < 0.01$.

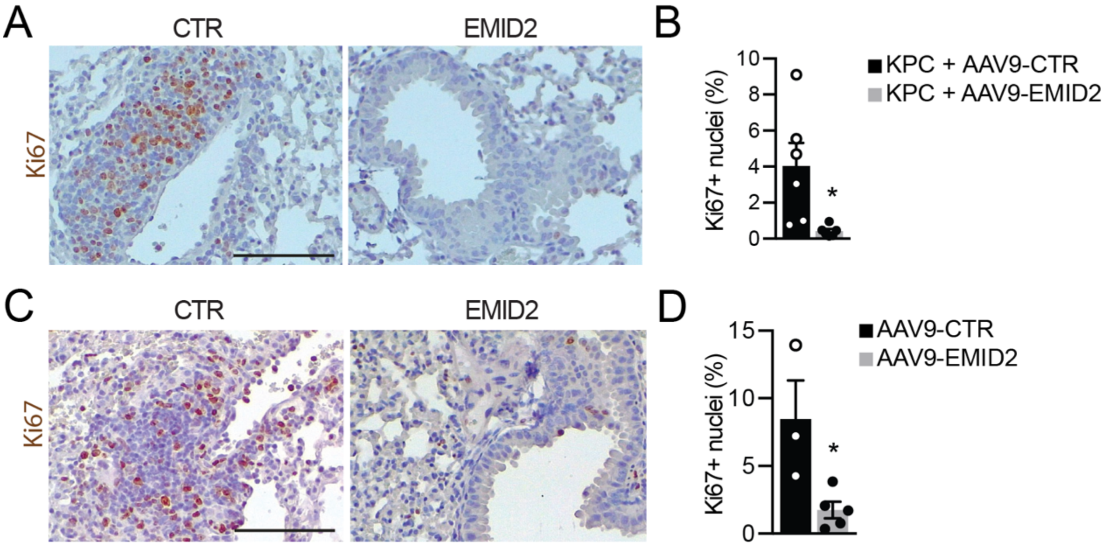

Supplementary Figure S8. A. Representative images of lung metastasis from KPC pancreatic tumors transduced with either AAV9-CTR or AAV9-EMID2, stained for Ki67. **B.** Quantification of $Ki67^+$ cells in lung metastasis. **C.** Representative images of lung metastasis from KC mice transduced with either AAV9-CTR or AAV9-EMID2, stained for Ki67. **D.** Quantification of $Ki67^+$ cells in lung metastasis. Scale bar in A, C, 0,100 µm. Data in B and D are shown as mean ± SEM and were analyzed by unpaired Student's T-test. *$p < 0.05$.

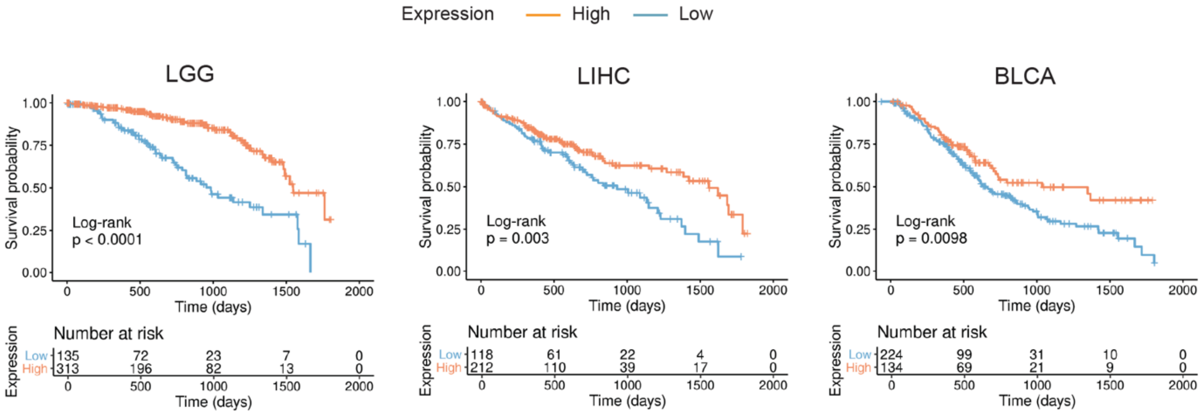

Supplementary Figure S9. Kaplan-Meier curves showing survival probability of patients affected by LGG, LIHC and BLCA, according to the expression levels of EMID2, at the indicated time points from diagnosis. The lower part of the panels indicates the number of samples included in each time point. Data source: TCGA database.

EXTENDED MATERIAL AND METHODS

Animal studies

Institutional guidelines in compliance with national and international laws and policies were followed for animal care and treatment. All experimental procedures were approved by the ICGEB Animal Welfare Board, with the requirements of the EU Directive 2010/63/EU, and by the Italian Ministry of Health (authorizations n. 539/2019-PR and 645/2019-PR).

CanSel screening. To select the most appropriate cell type for screening, B16-F10 melanoma cells (ATCC, #CRL-6475), LG lung adenocarcinoma cells, derived from Kras$^{G12D/+}$;Trp53$^{-/-}$ mice (1), and LLC lung adenocarcinoma cells (ATCC, #CRL-1642) cancer cells, were implanted into the *tibialis anterior* muscle of C57BL/6 mice, whereas 4T1 breast cancer cells (ATCC, #CRL-2539) into the *tibialis anterior* of Balb/c mice (50,000 cells per injection, n = 3 muscles/group). For the screening, each AAV9 pool was injected bilaterally in the *tibialis anterior* muscle of adult C57BL/6 mice (3 x 10^{10} viral genomes per muscle, 5 animals per pool). After one week, the same muscles were injected with 50,000 LLC cells (3 mice per pool). All muscles were harvested 10 days after cancer cell injection. In this way, each pool was injected in 6 muscles in combination with cancer cells, as well as in 4 additional muscles in the absence of cancer cells (controls). Total muscle DNA was extracted using the DNeasy Blood & Tissue Kit (Qiagen, #69504) for barcode amplification, using a validated two-step PCR protocol (2). Amplicons were finally analyzed by NGS, using an Illumina platform, to identify the relative abundance of each barcode, and thus of each transgene. To compare the relative abundance of transgenes belonging to different pools, we first calculated the mean log value of the frequency of each transgene in muscles injected with each AAV pool (*mean log control AAV*). Then, we assessed the log of the frequencies of the same transgene in every muscle injected with the same AAV pool and cancer cells (*log AAV LLC*) and normalized the relative abundance of each transgene, as *log AAV LLC: mean log control AAV ratio*. Column B in **Supplementary Table S2** shows the average value of this ratio for each transgene (*avlog*). *Avlog* values close to 0 indicate no enrichment, as expected for factors that do not influence cell invasion (grey area in graphs of **Supplementary Fig. S2B**). Factors that were enriched over 1SD were expected to exert anti-invasive effects (red area in **Supplementary Fig. S2B**). Factors that were not detected in either transduced muscles or the original viral preparation, were excluded from the analysis and are shown in orange in **Supplementary Fig. S2B**. Only transgenes with reads in at least three replicates were included in the analysis. To validate screening results, LLC (5 muscles/group) or LG cells (6 muscles/group) were injected to the *tibialis anterior* muscle of C57BL/6 or COLL-EGFP/SMA-RFP mice (3) together with 1.5 x 10^{10} vg of AAV vectors. Mice were sacrificed at 10 days.

Orthotopic model of lung adenocarcinoma. Adult C57BL/6 mice were locally injected with either AAV6.2FF-EMID2 or AAV6.2FF-CTR (2 x 10^{10} vg per mouse) into the left lung parenchyma through intercostal injection at the median axillary line. Two weeks later, 2.5 x 10^5 LG cells (1) were injected in the tail vein of the same animals (6 mice/group).

Orthotopic model of pancreatic adenocarcinoma. Pancreatic ductal adenocarcinoma cells derived from KPC (*LSL-Kras$^{G12D/+}$; LSL-Trp53$^{R172H/+}$; Pdx1-Cre*) mice (kindly donated by M. Martini and E. Hirsch, University of Torino) were dissociated into single cells with 0.25% trypsin (GIBCO), washed with serum-free Dulbecco's

Modified Eagle's medium (DMEM) twice, and counted in preparation for implantation into syngeneic mice. 8-week-old C57BL/6J mice were anesthetized with isofluorane and injected orthotopically with a suspension containing $1x10^5$ cells and AAV9-EMID2 or AAV9CTR (3×10^{10} vg per mouse). Animals were sacrificed 4 weeks after tumor challenge and pancreas and lungs were harvested and fixed in formalin for histological evaluation. Mouse and pancreas weights were also recorded (>3 mice/group).

Genetic model of pancreatic adenocarcinoma. To generate KC mice, *LSL-Kras^{G12D}* mice (Jackson Labs #008179) were bred with *Pdx1-Cre* mice (Jackson Labs #014647). Genotypes were confirmed by PCR amplification of ear snips digested with proteinase K, as previously described (4). Primers used were: JaxCre Fw - TGCCACGACCAAGTGACAGC; JaxCre Rw -CCAGGTTACGGATATAGTTCATG; Kras Fw - GTCTTTCCCCAGCACAGTGC; Kras Rv - CTCTTGCCTACGCCACCAGCTC; Kras mut – AGCTAGCCACCATGGCTTGAGTAAGTCTGCA. Mice were born according to expected Mendelian ratios and no exclusion parameters were applied. A mix of males and females was used for all experiments. Mice were injected with AAV9 (3×10^{10} vg per mouse) at 6 weeks of age and then treated with cerulein to induce acute pancreatitis and trigger pancreatic carcinogenesis (4). Mice were starved overnight, and then intraperitoneally injected with 12 hourly injections of cerulein (SIGMA #C9026; 100 µg/kg diluted in PBS) on two consecutive days. Mice weight and mobility were monitored every 3-5 days. Mice were sacrificed 4 weeks after cerulein injection; pancreas and lungs were harvested and formalin-fixed for histological analysis (>3 mice/group).

AAV vectors

Recombinant AAV vectors were produced by the AAV Vector Unit at ICGEB in Trieste (https://www.icgeb.org/facilities/avu-core-facility/). The cDNAs corresponding to the mouse secretome library were individually cloned into a modified pZac2.1 vector (pGi), under the control of the cytomegalovirus IE promoter, and used to produce the corresponding AAV9 vectors. At the 3' end of the coding sequence, a DNA barcode, consisting of a variable 10 nucleotide sequence, was inserted to uniquely identify each vector by Next Generation Sequencing (NGS). The viral preparations used in this work had titers higher than 10^{12} viral genomes/mL (5).

Barcode DNA Amplification and Next-Generation Sequencing

A unique 10–base pair sequence (barcode) was inserted at the 3' end of each cDNA, and specifically amplified with two subsequent cycles of polymerase chain reaction (PCR) amplification, first with primers annealing to a common region on the pGi vector and then with primers containing an Illumina index for NGS with HighSeq 2000 Illumina (IGA Technology Services, Udine). This procedure has been previously described and validated (2).

Primary fibroblast cultures

Primary fibroblasts were isolated from the abdominal skin of C57BL/6 and COLL-EGFP/αSMA-RFP (3). The skin was rinsed with calcium- and bicarbonate-free Hank's solution with HEPES (CBFHH) and incubated overnight with dispase II (5

mg/mL, Thermo Fisher Scientific). The tissue was further digested by an enzyme mixture composed by collagenase I (1 mg/mL, Worthington Biochemical Corporation) and DNase II (0.4 mg/mL, Sigma) for 1 hour at 37°C. The digested tissue was then diluted in Dulbecco's Modified Eagle Medium (DMEM, Sigma), filtered and centrifuged at 400 x g for 10 minutes.
Fibroblasts were isolated by plating the digested tissue suspension for 2 hours, followed by removal of non-adherent cells. Fresh DMEM supplemented with 10% FBS was added to attached fibroblasts.
For ECM production, fibroblasts were cultured for 9 days on glass coverslips, pre-treated with 0.2% gelatin, 1% glutaraldehyde and 1 M ethanolamine. Culture medium was changed daily and supplemented with ascorbic acid (50 µg/mL) and 1 µg/mL of recombinant mouse EMID2 (rEMID2, CusaBio, #CSB-BP820987MO). To decellularize the ECM, coverslips were incubated with a buffer composed by 0.5% Triton X-100, 20 mM NH_4OH in PBS (6). After washing, LG cells and NIH-3T3 fibroblasts (ATCC, #CRL-1658) were seeded on this matrix.

Cellular assays

Production of medium enriched for 10 selected factors. HEK293T cells (ATCC, #CRL-3216) were transfected with pGi plasmids carrying the cDNA encoding each secreted factor. Cells were cultured for 24 hours in FBS-free DMEM to collect supernatants enriched for each factor. Empty pGi plasmid was transfected to produce control supernatants.
Transwell assay. Cells (5 x 10^4 iMAEC or 10^5 LLC cells) were seeded on Transwell Permeable Supports (Corning, #3421) in serum-free medium, adding enriched supernatants to the bottom well. After an overnight incubation, the migrated cells were labelled with Hoechst.
Scratch assay. Human skin fibroblasts (15 x 10^3 cells) were seeded in 24-well plates and transfected with either a plasmid expressing EMID2 or an empty plasmid (pGi) using Fugene HD (Promega) for 7 hours in antibiotic-free DMEM supplemented with 10% FBS. At the same time, CellTracker Deep Red (ThermoFisher) was added to PANC-1 human pancreatic cancer cells (15 x 10^3 cells), suspended in PBS and incubated for 30 minutes at 37°C, 5% CO_2, protected from light. After incubation, cells were centrifuged for 5 min at 300 × g, the supernatant was discarded and the cells resuspended in DMEM supplemented with 10% FBS and antibiotics (penicillin/streptomycin, 100µg/mL). Labeled PANC-1 cells were seeded in the wells containing transfected human skin fibroblasts in the DMEM supplemented with 10% FBS and antibiotics (penicillin/streptomycin, 100µg/mL). After reaching a confluency of approximately 70–90%, a scratch was performed in the center of each well using a pipette tip (200µL size). Images were acquired immediately after the scratch and after 20 hours.

All cellular assays were performed in triplicates.

YAP localization

Matrigel (50µl) diluted 1:10 in PBS was allowed to polymerize at 37°C for 1 hour with or without rEMID2 (20µg/ml) in µ-Slide 8 Well Ibidi chambers (Ibidi, #80806). LG cells (5x10^3 cells/well) were seeded on Matrigel for 24 hours, then they were fixed in 4% PFA, permeabilized with 0.5% Triton X-100, blocked in 10% horse serum and incubated with anti-YAP primary antibody (1:100 Santa Cruz #101199).

RNA extraction and real-time PCR

Total RNA was isolated using TRIzol (Invitrogen). Reverse transcription was performed on 1 µg of total RNA using First Strand cDNA Synthesis Kit (Thermo Scientific) with random hexameric primers. The expression of *Ctgf, Cyr61, c-myc, Axl* and 18S RNA was quantified using SYBR Green and the following primers:
Ctgf F: TGCCTGTTTAAGCAGAAAGCTG-3', R: TGAGCACACTAAATAAGGCTTTCA-3'
CYR61 F: AACCCATCCAGGCAATCAGG-3', R: GACACATAGTAGGTCCTCAGGG-3'
c-myc F: AAAACGACAAGAGGCGGACA-3', R: ATTCAGGGATCTGGTCACGC-3'
Axl F: ATTCGGTTTCCCCACTGTGA-3', R: CACGATGGCCTGCAACTAAC-3'
18S F: GGC CCT GTA ATT GGA ATG AGT-3', R: CCA AGA TCC AAC TAC GAG CTT-3'

Histology and immunostaining

Histology and immunofluorescence were performed on fixed cells and tissues. Cells on plastic were fixed with 4% PFA, whereas cells were fixed with 0.1% PFA for 1 hour. Tissue samples were either snap-frozen or embedded in paraffin and cut into 5 µm slices.
For immunofluorescence sections were permeabilized in Triton X-100, blocked in 10% horse serum and incubated with either Alexa Fluor 488 Phalloidin (1:500 ThermoFisher Scientific #a12379) or primary antibodies, targeting collagen I (1:200 Abcam #ab21286), fibronectin (1:200 Abcam # ab2413), Ki67 (1:200 Cell Signaling #9129), laminin (1:500 Sigma-Aldrich #L9393), paxillin (1:100 Abcam #ab32084) and alpha-Smooth Muscle Actin-Cy3 (1:300 Sigma-Aldrich #C6198). Alexa Fluor-conjugated secondary antibodies from Invitrogen were used to detect primary antibodies, while nuclei were counterstained with Hoechst.

For immunohistochemistry, 5 µm paraffin sections were deparaffinized, rehydrated and antigen retrieved was performed in a calibrated steam pressure cooker with citrate buffer (pH 6.0). Endogenous peroxidase was blocked 3% H_2O_2 in PBS for 10 minutes. The slides were rinsed in 0.1% Tween-20 in PBS and blocked with 5% nonfat dry milk for 20 minutes to minimize nonspecific binding. The slides were then incubated in blocking buffer with mouse anti-YAP antibody (Santa Cruz, sc-101199, 1:100) overnight at 4°C in a humidified chamber. After washing with 0.1% Tween-20, in PBS, slides were incubated with goat anti-mouse antibody conjugated to HRP (Abcam ab214879) for 30 minutes and washed in PBS. Colorimetric detection was performed using 3-3'-Diaminobenzidine chromogen and hydrogen peroxidase (DAB Quanto, Epredia) for a few minutes and counterstained with hematoxylin (Sigma-Aldrich, St. Louis, MO, USA).

Microscopy and image analysis

Images were acquired with a Leica microscope equipped with a DFC300 camera and LAS V4.4 software, a Nikon A1 Plus microscope equipped with DC-152Q-C00-FI and NIS 4.30 software (Nikon) and a ZEISS LSM 880 with Airyscan.
At least 6 images per sample were considered for both *in vitro* and *in vivo* experiments. Individual values shown in graphs represent the mean of all quantifications referring to the same biological replicate.

Images were analysed using ImageJ2 (Fiji) software. For analysis of circularity, the tumor border was drawn by free-hand tool to measure both perimeter (P) and area (A), followed by calculation of circularity (C) according to the following equation: $C = 4\pi (A/P^2)$. The circularity index was then calculated as the average of biological replicates.

Bioinformatic analysis

Gene Ontology was performed using the Cytoscape (v. 3.9.1) plug-in ClueGO (v. 2.5.8) (7,8), which estimates the pathways enrichment score on pre-selected set of genes, exploiting a two-sided hypergeometric test. The Gene Ontology Biological Processes (GO-BP) database has been selected as reference (9). Pathways with less than 4 associated genes from the uploaded gene list were discharged. Functional related GO terms were grouped by setting a similarity threshold of kappa score of 0.4. Pathways with an associated p-value < 0.05 were deemed as significant.

Survival analysis was performed using TGCA survival tool (TCGAsurvival: scripts to extract TGCA data for survival analysis https://github.com/mdozmorov/TCGAsurvival accessed march 2021) implemented in R/Bioconductor Environment. Briefly, the steps consisted in downloading the data from the repository, performing standard coxph models for each tumor and plotting the results using survminer package and forestplot package.

Statistical analysis

Statistical analyses were performed using GraphPad Prism 7.0 on data expressed as mean ± standard error. Statistical significance was determined using unpaired Student's T-test and one-way ANOVA with Student-Newman-Keuls correction. A P value lower than 0.05 was considered significant (*$p < 0.05$, **$p < 0.01$, ***$p < 0.001$).

References

1. Dimitrova N, Gocheva V, Bhutkar A, Resnick R, Jong RM, Miller KM*, et al.* Stromal Expression of miR-143/145 Promotes Neoangiogenesis in Lung Cancer Development. Cancer Discov **2016**;6:188-201
2. Bortolotti F, Ruozi G, Falcione A, Doimo S, Dal Ferro M, Lesizza P*, et al.* In Vivo Functional Selection Identifies Cardiotrophin-1 as a Cardiac Engraftment Factor for Mesenchymal Stromal Cells. Circulation **2017**;136:1509-24
3. Magness ST, Bataller R, Yang L, Brenner DA. A dual reporter gene transgenic mouse demonstrates heterogeneity in hepatic fibrogenic cell populations. Hepatology **2004**;40:1151-9
4. Carrer A, Trefely S, Zhao S, Campbell SL, Norgard RJ, Schultz KC*, et al.* Acetyl-CoA Metabolism Supports Multistep Pancreatic Tumorigenesis. Cancer Discov **2019**;9:416-35
5. Ruozi G, Bortolotti F, Falcione A, Dal Ferro M, Ukovich L, Macedo A*, et al.* AAV-mediated in vivo functional selection of tissue-protective factors against ischaemia. Nat Commun **2015**;6:7388

6. Franco-Barraza J, Beacham DA, Amatangelo MD, Cukierman E. Preparation of Extracellular Matrices Produced by Cultured and Primary Fibroblasts. Curr Protoc Cell Biol **2016**;71:10 9 1- 9 34
7. Shannon P, Markiel A, Ozier O, Baliga NS, Wang JT, Ramage D*, et al.* Cytoscape: a software environment for integrated models of biomolecular interaction networks. Genome Res **2003**;13:2498-504
8. Bindea G, Mlecnik B, Hackl H, Charoentong P, Tosolini M, Kirilovsky A*, et al.* ClueGO: a Cytoscape plug-in to decipher functionally grouped gene ontology and pathway annotation networks. Bioinformatics **2009**;25:1091-3
9. Gene Ontology C. Gene Ontology Consortium: going forward. Nucleic Acids Res **2015**;43:D1049-56
